# Application of the force-field technique to drought vulnerability analysis: A phenomenological approach

**DOI:** 10.4102/jamba.v11i1.589

**Published:** 2019-03-13

**Authors:** Bernard M. Hlalele

**Affiliations:** 1 Department of Business Support Studies, Central University of Technology, Bloemfontein, South Africa

**Keywords:** Force-field technique, phenomenology, vulnerability, drought, hazard, disaster

## Abstract

Drought events are integral parts of climate variability. Over 80% of the Basotho population’s livelihood is dependent on rain-fed agriculture. This current study used force-field project management technique for drought vulnerability analysis. The study was approached from a phenomenological viewpoint conceptualised using a force-field analysis technique. The study used a qualitative research approach and a phenomenological research design. The main findings of this study were high unemployment rate, environmental degradation and poverty. The study proposes that post- education outreaches and community capacity-building projects should be effected. This can take several forms, such as learnerships, where everyone is certificated for the job trained for. The study proposes that all registered institutions in country, be it catering, Basotho Enterprises Development Corporation (BEDCO), construction companies, driving schools, non-governmental organisations (NGOs), private companies, driving schools, NGO’s, private companies and so on, can take part in the training of members of the community in partnership with well-known institutions such as the National University of Lesotho, St, Elizabeth, Lerotholi Polytechnic and Limkonkwin Creative University for accredited informal certification. This skilled labour force can then be trained on how to create and form cooperatives to assist the government with the task of employment. Ha Masupha and Ha Thakanyane are places that are less advantaged with poor transport and road infrastructure and high migration rates and poverty levels. If all or a majority of the proposed actions for change are effected, there will be fewer new HIV/AIDS infections, improved quality of life, less deforestation caused by poor socio-economic status and less graduate unemployment for all Thabana Morena communities.

## Introduction

Disasters have the potential to destroy in a very short period development inputs that have been made over a number of years, and they can also delay future development because of loss of resources. These resources may need to be shifted to places of emergencies for response, thereby retarding investment (Reed 1997). Development, on the other hand, can increase vulnerability to disasters: for instance, when developments occur, population density increases, thereby increasing development of hazardous sites, environmental degradation, technological failures and imbalance of pre-existing natural or social systems (Reed 1997). However, disasters not only have negative effects but can also create a platform for new developments, in that after disasters new buildings that adhere to building codes may be erected, hence creating a political and social atmosphere of acceptance for change. International aid is focused on the areas affected by disasters, which finally results in development. In Kenya, after prolonged drought periods in the 1970s and 1980s, national and international efforts were directed towards former pastoralist populations in Marsabit District by organisations such as the Catholic Church and Africa Inland Mission in settling nomads and developing small towns, while international efforts were effected by United Nations Educational, Scientific and Cultural Organization’s (UNESCO’s) Integrated Project in Arid Lands through focusing on a range of conservation efforts and improvement of livestock marketing. The sedentarisation of pastoralists led to better access to education, healthcare and other social services, as well as contributing to rural proletarianisation and economic differentiation. Currently in Kenya the local economies are a combination of subsistence pastoralism, livestock marketing and wage labour, showing the process of sedentarisation (Fratkin 2007). In a study by Peduzzi et al. (2009) on global exposure and vulnerability towards hazards in which drought was amongst the selected hazards, it was found that human vulnerability had a direct link with a country’s development and the quality of the environment. In Lesotho, the British Red Cross, in collaboration with the Lesotho Red Cross, provided help to Basotho who were in desperate need of food; such assistance lasted for 2 days, in four districts in which Mafeteng district was one of the districts affected (British Red Cross 2013). One of the Lesotho government’s development goals, laid out in *Lesotho’s Vision 2020*, is that Lesotho shall have a healthy and well-developed human resources base, a well-managed environment, a strong economy as well as an established technological base. In his inaugural speech, Prime Minister Thomas Thabane affirmed that the government would commit itself to the goals as set out in the National Strategic Development Plan, two of which were the reduction of vulnerabilities and reversing environmental degradation and adapting to climate changes (World Bank 2014). From this it can be deduced that Lesotho focuses on short-term responses rather than seeking a permanent or close to permanent solution to disaster response, which in turn may result in social development, through creation of jobs and other economic activities for sustainable development like countries such as Kenya. Drought disasters lead to both water and food shortages, which ultimately are likely to have adverse effects on the economy, environment and health of the population (WHO 2014). The following are factors that influence drought impacts: demographic pressure on the environment, food insecurity, agricultural dependent economic systems, poor infrastructure (irrigation, water supply and sanitation systems), poor health status of communities before disasters, time of the year (the most critical being before the harvest), lack of warning systems, population displacement and other concurrent situations such as political instability, economic crises and conflicts (WHO 2014). According to Reed (1997), drought is more severe in dry areas that have a limited amount of rainfall. There are also physical factors, such as soil moisture retention and timing of the rain, that influence the degree of crop loss during drought periods. Vulnerability is also increased by dependency on rain-fed agriculture, livestock-dependent communities with limited grazing territories and exhaustion of coping mechanisms, which may lead to population displacement. Similarly, the United Nations Framework Convention on Climate Change (UNFCCC) (2007) states that there is a lack of adaptive capacity and stable prosperous economy to respond to natural hazards in the least developed countries. In a study undertaken by Swain and Swain (2011) in Western Orissa in India, it was found that factors that contributed to drought vulnerability were, amongst others, categorised into biophysical and socio-economic including rainfall variability, drought intensity and lack of water-holding capacity of soils; while socio-economic includes low irrigation development and poor crop insurance coverage. The study again showed that there were lower coping capacity levels compared to levels of drought risk and vulnerability. Similarly, Lesotho is said to be chronically food aid dependent, with over 80% of the population dependent on rain-fed agriculture for their livelihoods. The Department of Social Welfare provides assistance to most vulnerable groups in the country, in which regular food transfers are made to school children, expectant mothers and lactating mothers, terminally ill individuals and chronically food-insecure people. This has been going on for a period of over 20 years (UNDP 2014). Though vulnerability factors from various regions are almost the same, Lesotho’s drought vulnerability contributing factors can be summarised as poverty levels, health and well-being of individuals and dependency on rain-fed agriculture. Moreover, drought and desertification are serious challenges and threats that are facing sustainable development in Africa, and these have far-reaching negative consequences on human health, food security, economic activities, physical infrastructure, natural resources and the environment (UNECA 2008). In 2011, the Horn of Africa needed humanitarian assistance following a drought that affected 13 million people (Action Aid 2011). Lesotho has not been an exception and although Lesotho’s per capita income has increased, poverty is still one of the major challenges facing this country, which is attributed to the adverse effects of drought on agricultural production because agriculture is the backbone of Lesotho’s economy (African Development Bank Group 2013). In a report by the Lesotho Department of Planning (2008) on the Compilation of Crucial Information for the Mafeteng District, Koti-Se-Phola was the community council with the highest percentage, namely 7.8%, of people that needed food aid (Lesotho Department of Planning 2008). This council has 2754 households with an average size of seven people per household. In terms of the number of the households that have agricultural plots, it is the third with 2158 in total and with an average plot of 1.6 hectares per household (Lesotho Department of Planning 2008). This council is one of the largest in terms of agricultural land ownership, which when hit by drought is likely to affect people in large numbers. The current study aimed at assessing drought vulnerability in Koti-Se-Phola, Mafeteng, Lesotho, using qualitative methods of research in order to provide relevant decision-makers and NGOs with information; and to determine groups at risk and the coping mechanisms of communities and to suggest strategies to improve on the current coping mechanisms for drought. In addition to this, FAO (2011) shows that drought is considered the most severe cause of food shortages in developing countries; most reports and research in Lesotho indicate Mafeteng as a poor district and the most vulnerable to climate change in the country. However, none of these studies are drought-specific and approach vulnerability assessment in this district from community council level. Vulnerability is said to be dynamic in nature, varying from one place to another over a specific period of time (Birkmann 2006). In recent years, particularly 2014–2015, Lesotho was struck by a drought disaster that left thousands of Basotho in hunger and food insecurity. The majority of Lesotho nationals depend on rain-fed agriculture for their livelihood, which leaves them stranded during drought events. In the light of this, the current study assessed the driving and restraining forces to drought vulnerability at the Koti-Se-Phola community council for better government decision-making surrounding development. Moreover, results from this study can be used by government and authorities in planning against drought and building of community resilience to drought. A study conducted by Belle and Hlalele (2015) indicated that the Koti-Se-Phola community council was vulnerable to damaging drought effects.

## Study area description

**FIGURE 1:**
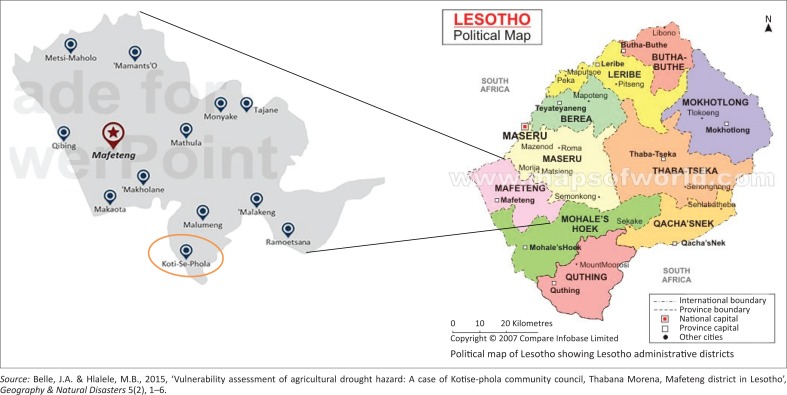
Mafeteng district community councils.

Lesotho is a lower–middle-income country comprising an area of 30 000 km^2^ and it is ranked number 158 out of 186 countries according to the 2012 UNDP Human Development Index (WFP 2013). Lesotho is said to be one of the most vulnerable countries to drought, with Mafeteng being one of the districts that is hardest hit by prolonged drought and erratic seasonal rainfall patterns (WFP 2013). Lesotho is divided into ten administrative districts: Maseru, Berea, Leribe, Butha-Buthe, Mokhotlong, Thaba-Tseka, Qacha’s Nek, Quthing, Mohale’s Hoek and Mafeteng (Figure 1). Moreover, Lesotho is further categorised into four distinct agro-ecological zones: the lowlands, foothills, mountains and Sengu River Valley. These zones are characterised by distinct differences in climatic and ecological conditions. Mafeteng district comprises mainly lowlands and only a small portion consists of foothills. In these zones, the top soil is sandy and susceptible to both wind and water erosion because of overgrazing (BOS 2010). Lesotho has a temperate climate with very cold winters and hot summers. Temperatures get down to −7 °C in the lowlands in winter. The yearly precipitation is between 600 mm and 1200 mm in the lowlands whereas the annual precipitation in the country is between 700 mm and 800 mm. This large variance in rainfall leads to periodic droughts (BOS 2010). The districts are further subdivided into 128 district councils. Mafeteng district is subdivided into 12 community councils, namely Koti-Se-Phola, Makaota, Makholane, Malakeng, Malumeng, Mamantsi’O, Monyake, Mathula, Metsi-Maholo, Qibing, Ramoeletsi and Tajane. Koti-Se-Phola is found in the south of this district and is partly lowlands and foothills. Within this community council area, there is the Makhaleng River, which runs in close proximity to Maholong and Ha Masupha villages. The secondary school enrolment in Mafeteng district in the years 2008, 2009 and 2010 stood at 10.4%, 11.4% and 10.0%, respectively (BOS 2010). In 2011–2012 the unemployment rate in the second quarter for Mafeteng district was 16.1% for people aged 15–64 years (BOS 2013). The majority of the communities in Lesotho depend on agriculture for a living, which when hit by drought leaves communities in food-insecure conditions. Koti-Se-Phola is made up of 41 villages from which the study sampled information (Lesotho Department of Planning 2008). Mafeteng district has a population of about 192 977, out of which the Koti-Se-Phola community council comprises 12 393. In this community council, there are 6119 and 6274 men and women, respectively. Of this population, 7.8% receive food aid. Members of the community receive agricultural support from the Ministry of Agriculture and NGOs (Red Cross, World Vision and Catholic Relief Services). In terms of trade and commerce, there are 48 cafes, two supermarkets and six bars, with no banking facilities (Lesotho Department of Planning 2008). Figure 1 shows the location of the study area.

## Methods and materials

*Phenomenology* is defined as an approach to qualitative research that focuses on the commonality of lived experiences within a particular group (Maxwell 2013). The main aim of this approach to research is to provide a full description of the nature of the phenomenon in question (Creswell 2013). One of the main advantages of phenomenological research is that it does not call for complex and sophisticated technologies for data collection and analysis but relies on one’s lived experiences (Schutz 1970; Waters 2017). Moreover, this research is used in collaboration with force-field analysis. Force-field analysis is a powerful tool for building an understanding of the forces that drive and resist a proposed change (SkyMark Coorporation 2017). This systematic data analysing tool is used in complex problem situations. The problem is framed in terms of factors or pressures that support the status quo (restraining forces) and those pressures that support change in the desired direction (driving forces) (Thamas 1985). In this study, the force-field was used as the conceptual framework approached from a phenomenological viewpoint.

## Results and discussions

This section presents all forces for and against the desired change for community development in the study to counter the adverse effects of drought events. The current study ends with those forces that need to be monitored in order to reach a desired change, all from the researcher’s lived experience. The researcher lived in this place from childhood until the age of 27. The researcher was once a shepherd and a scholar simultaneously, looking after his father’s flock, which included cattle, ewes, rams, goats, horses and donkeys. These animals were grazed over all the pastures in the Thabana Morena region in which the Koti-Se-Phola community falls. The sheep the researcher looked after were later used as the means to pay for his tuition fee at Mt Tabor High School during the tenure of Principal David Kiwanuka. The study area is plagued with high poverty levels, HIV prevalence, unemployment, migration, stock theft and overgrazing problems; hence, land degradation has resulted in a network of dongas across fields and pastures.

### The desired change

The current study aims to describe a plan for sustainable drought-resilient communities in Thabana Morena (constituency number 52) in Mafeteng, Lesotho, including improvement of quality of life through creation of off-farm activities for livelihood means.

### Driving forces

**FIGURE 2:**
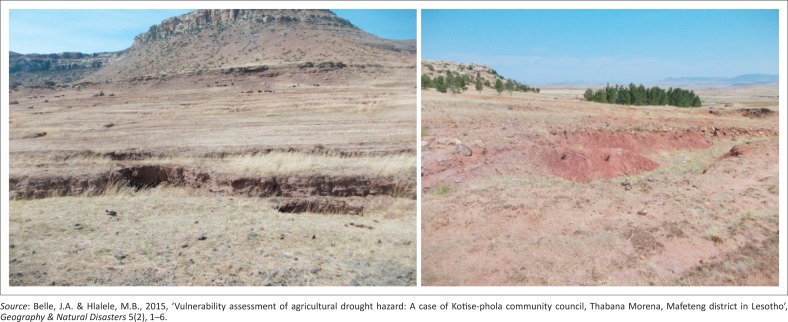
Eroded fields between Ha Ngoae, Khubetsana and Maralleng.

According to Belle and Hlalele (2015), this area is plagued with high levels of unemployment and large household sizes, which make it difficult to cope during drought events. Hlalele (2017a) asserts that in addition to the current lack of rainfall, there is poor rangeland management, which has led to the formation of networks of dongas across crops fields; hence, there are land degradation problems in the area. The majority of households in Thabana Morena use wood as the main source of energy for both cooking and warming in winter. The situation has been exacerbated by land exposure to both wind and water, resulting in soil erosion. The areas that are exposed to wind and water erosion are frequented by drought events (Hlalele 2016). Another factor that drives community vulnerability to drought events is the culture and tradition, where boys are expected to collect and gather logs and tree trunks on a daily basis as part of their initiation culture. This situation, however, has left the environment vulnerable to long-term drought episodes. Figure 2 shows some of the eroded crop fields between Ha Ngoae, Khubetsana and Maralleng. Poverty is another factor that is driving young community members out into illegal mining in old, closed South African mines. From 2011 to 2017, nine young men from Thaba-Chitja (Ha Thakanyane, Lehana, Masupha, Thabaneng and Maholong) who left their homes for this illegal activity lost their lives (Hlalele 2017b).

**FIGURE 3:**
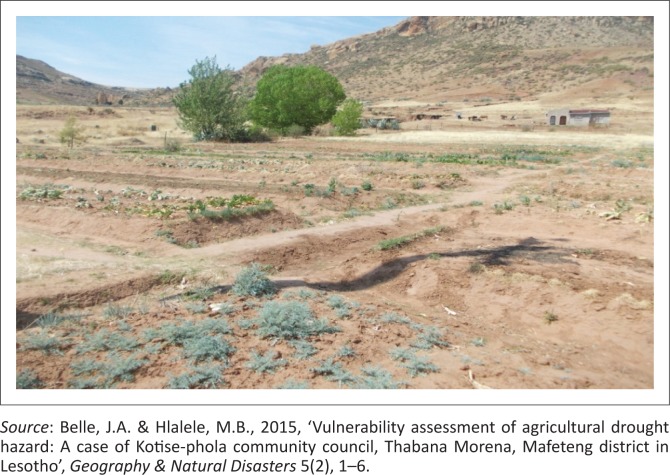
Abandoned community vegetable project at Ha Ngoae.

Several poverty alleviation projects have been set up in the region, including poultry and community vegetable projects in some of the villages in Thabana Morena such as Ha Ngoae. Figure 3 shows an abandoned community vegetable project at Ha Ngoae.

**FIGURE 4:**
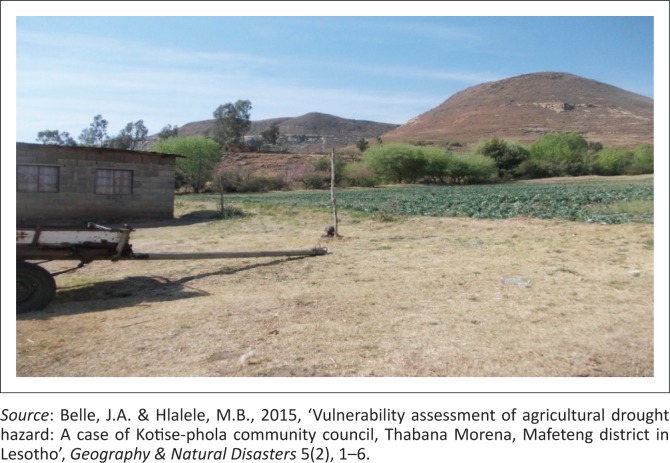
Mr Matea’s cabbage field at Ha Bofihla, Thabana Morena (2014–2015).

The high unemployment situation in this area has resulted in some individual subsistence farmers such as Mr Matea at Ha Bofihla ploughing a cabbage field to earn a livelihood through small-scale cabbage selling. Figure 4 shows Mr Matea’s cabbage field watered from a nearby water source. The human immunodeficiency virus and *acquired immune deficiency syndrome* (HIV and AIDS) are factors that have made this community more vulnerable to drought effects, where the elderly and the younger groups are left alone in homes for urban areas. HIV-infected individuals, the young and the elderly, find it difficult to cope with water collection and health issues as a result of lack of food, which compromises their immune systems.

### Restraining forces

As indicated, the force-field analysis identified forces for and against the phenomenon in question; this section pinpoints the forces that prevent the Thabana Morena communities from moving towards sustainable drought-resilience. According to the researcher, the major restraining forces for sustainable development and resilience to drought disasters are lack of education, lack of employment and political will in disaster and development issues. Thousands of Basotho have left the country for South Africa because of lack of job opportunities in Lesotho. Most of these people were initially the uneducated, who worked on farms and as nannies. However, the situation is far worse than in the past, where even graduates have to settle for nanny jobs in South Africa. It must be noted that this is only the fortunate group in the country; a majority of these graduates are in their second through sixth year of unemployment, included but not limited to one Bachelor of Commerce graduate seen on social media dressed in academic regalia selling ice cream in Lesotho. The situation calls for urgent attention by those in authority to capacitate their people and mitigate negative effects in drought events and other sister disasters in the country. Moreover, there is poor rangelands management complemented by lack of law enhancement with regard to environmental protection where people cut trees in their home’s vicinity freely.

## Conclusion and recommendations

This study ends the discussion of the two opposing forces with a comprehensive change strategy viewed from the force-field analysis conceptual framework. In order to move these communities towards the desired direction, the study proposes that education outreach be conducted where members of the communities know the consequences of not protecting the environment. This is not enough and may mean nothing if unemployment issues are not addressed. After education outreach, community capacity-building projects can take place. This can take several forms such as leaderships where everyone will be trained and certified for a job. The study proposes that all registered institutions, be it catering, Basotho Enterprises Development Corporation (BEDCO), construction companies, driving schools and so on, can train members of the community in partnership with well-known institutions such as the National University of Lesotho, St Elizabeth, Lerotholi Polytechnic and Limkonkwin Creative University for accredited informal certification. These skilled human resources can then be trained on how to create and form cooperatives to assist the government with the task of employment. The best way to do this is to decentralise resources far down to Ha Masupha and Ha Thakanyane. These are less advantaged places with poor transport and road infrastructure and a high migration rate. If all the proposed actions for change are effected, there will be lower HIV/AIDS rates, improved quality of life, less deforestation caused by poor socio-economic status and less graduate unemployment for all Thabana Morena communities.
